# The impact, cost and cost‐effectiveness of oral pre‐exposure prophylaxis in sub‐Saharan Africa: a scoping review of modelling contributions and way forward

**DOI:** 10.1002/jia2.25390

**Published:** 2019-09-19

**Authors:** Kelsey K Case, Gabriela B Gomez, Timothy B Hallett

**Affiliations:** ^1^ Department of Infectious Disease Epidemiology Imperial College London London UK; ^2^ Department of Global Health and Development London School of Hygiene and Tropical Medicine London UK

**Keywords:** PrEP, modelling, combination prevention, cost‐effectiveness, sub‐Saharan Africa

## Abstract

**Introduction:**

Oral pre‐exposure prophylaxis (PrEP) is a new form of HIV prevention being considered for inclusion in national prevention portfolios. Many mathematical modelling studies have been undertaken that speak to the impact, cost and cost‐effectiveness of PrEP programmes. We assess the available evidence from mathematical modelling studies to inform programme planning and policy decision making for PrEP and further research directions.

**Methods:**

We conducted a scoping review of the published modelling literature. Articles published in English which modelled oral PrEP in sub‐Saharan Africa, or non‐specific settings with relevance to generalized HIV epidemic settings, were included. Data were extracted for the strategies of PrEP use modelled, and the impact, cost and cost‐effectiveness of PrEP for each strategy. We define an algorithm to assess the quality and relevance of studies included, summarize the available evidence and identify the current gaps in modelling. Recommendations are generated for future modelling applications and data collection.

**Results and Discussion:**

We reviewed 1924 abstracts and included 44 studies spanning 2007 to 2017. Modelling has reported that PrEP can be a cost‐effective addition to HIV prevention portfolios for some use cases, but also that it would not be cost‐effective to fund PrEP before other prevention interventions are expanded. However, our assessment of the quality of the modelling indicates cost‐effectiveness analyses failed to comply with standards of reporting for economic evaluations and the assessment of relevance highlighted that both key parameters and scenarios are now outdated. Current evidence gaps include modelling to inform service development using updated programmatic information and ex post modelling to evaluate and inform efficient deployment of resources in support of PrEP, especially among key populations, using direct evidence of cost, adherence and uptake patterns.

**Conclusions:**

Updated modelling which more appropriately captures PrEP programme delivery, uses current intervention scenarios, and is parameterized with data from demonstration and implementation projects is needed in support of more conclusive findings and actionable recommendations for programmes and policy. Future analyses should address these issues, aligning with countries to support the needs of programme planners and decision makers for models to more directly inform programme planning and policy.

## 1 Introduction

Sub‐Saharan Africa (SSA) remains a region disproportionately affected by HIV. It is estimated that nearly 26 million people in this region are currently living with HIV with 1.2 million new infections occurring annually 1. Given this substantial burden, efforts and investments are ongoing to continue to develop and expand the available HIV prevention options. Oral pre‐exposure prophylaxis (PrEP), the prophylactic use of antiretroviral drugs to prevent acquisition of HIV infection, is a relatively new prevention strategy currently being considered for inclusion in combination HIV prevention portfolios. This follows the initial demonstration of the effectiveness of oral PrEP among men who have sex with men (MSM) in the iPrEX trial 2. Since then, numerous trials have published mostly positive results across different population groups 3, 4, 5, 6, 7, 8. A consistent message across studies is that PrEP is effective when taken as directed 9.

Mathematical modelling which illustrates the epidemiological impact, budget impact and value for money of different PrEP strategies and assesses the importance of factors like uptake and adherence on population level impact, can be used to inform the adoption and implementation of national policy for PrEP. Considerable PrEP modelling has occurred over time with reviews of modelling summarizing that PrEP could be cost‐effective in SSA, particularly when appropriately prioritized to those at greatest risk of infection 10, 11, 12. However, much of the early modelling occurred at the same time as the PrEP efficacy trials and were exploratory in nature without programmatic evidence. In parallel, there has been rapid scale‐up of HIV treatment and prevention programmes, and evolving guidance and norms for cost‐effectiveness analyses.

With countries moving towards the adoption and implementation of national PrEP policies 13, and PrEP pilot and demonstration projects underway, there is an opportunity for mathematical modelling to inform priority setting and help decision makers designing implementation strategies. Therefore, we conducted a scoping review to assess the available modelling evidence for the impact, cost and cost‐effectiveness of oral PrEP in SSA at the time policy decisions for PrEP were being made. The specific objectives were to, (1) assess the quality and relevance of the available modelling evidence to inform policy in light of recognized best practices today, (2) identify the current technical and data gaps in PrEP modelling. The findings are then used to generate recommendations to inform future modelling and data collection, with the overall aim for modelling to better inform PrEP programme planning and policy decision making in SSA.

## 2 Methods

We conducted a scoping review of published literature adhering to the PRISMA extension scoping review guidance (see Additional File S1: PRISMA‐ScR checklist). We searched PubMed – a database broadly accessible to policy makers in sub‐Saharan Africa – using broad search criteria which consisted of permutations of the search terms *modelling*,* PrEP* and *HIV* (see Additional File S2 for detailed search strategy). No restrictions were placed on publication date and all published research was included up to the final search conducted 30 November 2017. Experts publishing in the field were consulted for search completeness. Articles published in English which modelled oral PrEP in sub‐Saharan Africa were included. Oral PrEP modelling in non‐specific settings with relevance to generalized HIV epidemic settings were also included. Studies were excluded if they focused on oral PrEP use in high‐income settings or in low‐ and middle‐income settings outside SSA, if they focused solely on modelling the pharmacokinetics or pharmacodynamics of oral PrEP or were focused exclusively on topical PrEP. Highly theoretical applications which focused on exploring the sensitivity of model and parameter assumptions and did not quantify the impact, cost or cost‐effectiveness of PrEP were also excluded. Review articles were not included, but their bibliographies were examined for search completeness. Letters to the editor were not included but correspondence that conducted modelling analyses, or reviewed and analysed conflicting modelling analyses, were examined to identify any issues or discrepancies in the published modelling results. The first author conducted the review, data extraction and assessment. Both co‐authors reviewed all results for consistency and resolved any discrepancies or queries that arose during this process.

### 2.1 Data extraction

The data extracted from each study included the study setting, population, modes of HIV transmission, timeframe of analysis, the key assumptions for PrEP efficacy and effectiveness, PrEP cost, ART cost, the question(s) and outcome(s) of interest, the scenarios investigated and a summary of the main findings. The specific questions of interest and the scenarios investigated in each modelling analysis were used to define potential strategies of PrEP use in countries in SSA. These strategies were organized by the relevant broader population group of interest – for example, strategies of PrEP use amongst the general population, serodiscordant couples, women, and men. Additionally, we compiled resource optimization strategies which investigated the potential role of PrEP in combination prevention with different levels of resource availability.

The modelling results for the impact (infections averted, reductions in incidence), cost (either financial or economic), and value for money (cost per infection averted, cost‐effectiveness) of oral PrEP were summarized for each strategy defined. The cost‐effectiveness measures of oral PrEP included the cost or incremental cost‐effectiveness ratio (ICER) per disability‐adjusted life year (DALY) averted, life year saved (LYS), or quality‐adjusted life year (QALY) saved, using thresholds of one to three times gross domestic product (GDP) per capita, as reported in the studies. We additionally defined an alternative cost‐effectiveness threshold of 0.5 times GDP per capita and evaluated the study results against this arguably more representative threshold 14, to identify the oral PrEP strategies which remain cost‐effective at this reduced threshold.

### 2.2 Assessment of evidence

An algorithm was defined to assess the relevance and quality of the available modelling evidence to inform policy. The evidence was assessed for each strategy of PrEP use. The results are represented using a traffic light system with the following assessment criteria applied:

- **Green**:
  1. Current: Use of currently appropriate assumptions and scenarios (e.g. PrEP efficacy, ART eligibility criteria) 15, 16
  2. Cost‐effectiveness analysis compliance: Cost‐effectiveness analyses comply with the Gates Reference Case 17
  3. Study agreement: Two studies with similar results
  4. Rigour: High rigour across modelling analyses based on criteria recommended for use to evaluate the quality of modelling defined by Garnett, *et al*. 18
- **Amber**: Failure for at least one of the above criteria
- **Red**: Failure across all criteria

The available modelling evidence across each strategy of PrEP use was assessed using this algorithm. Additional File S2 provides the specific criteria used to assess the cost‐effectiveness analysis compliance and rigour categories.

## 3 Results and discussion

**Figure 1:**
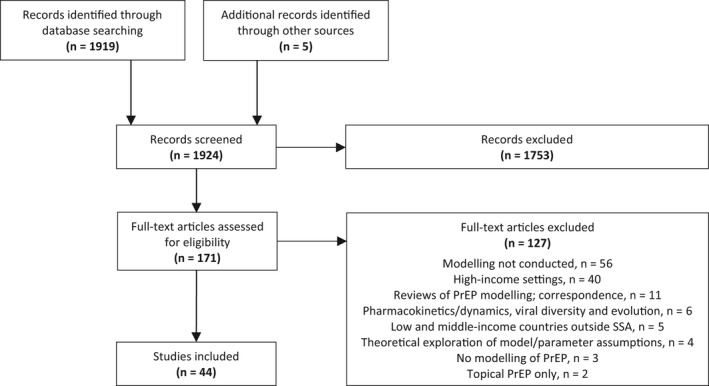
Flow diagram of study selection and the number of articles retrieved, screened, assessed for eligibility, and included and excluded. PrEP, pre‐exposure prophylaxis; SSA, sub‐Saharan Africa.

We reviewed 1924 abstracts and included 171 articles to review in full. The abstracts excluded at this stage were largely results from PrEP clinical trial studies and studies investigating knowledge, awareness, acceptability and uptake of PrEP. Of the 171 full‐text articles reviewed, 44 met the eligibility criteria and were included (Figure 1). These studies span from 2007 to 2017 with 42 studies 19, 20, 21, 22, 23, 24, 25, 26, 27, 28, 29, 30, 31, 32, 33, 34, 35, 36, 37, 38, 39, 40, 41, 42, 43, 44, 45, 46, 47, 48, 49, 50, 51, 52, 53, 54, 55, 56, 57, 58, 59 modelling oral PrEP in sub‐Saharan Africa and two studies 60, 61 with non‐specific settings but of relevance for generalized HIV epidemics. Applications of country‐specific modelling of oral PrEP occurred predominantly for South Africa 21, 23, 24, 28, 29, 31, 32, 35, 36, 38, 39, 41, 44, 48, 50, 54, 57, 59, 62, 63 and Kenya 25, 26, 30, 33, 34, 56, followed by Botswana 22, 52, 56, 62, Zambia 46, 47, 54, and one application each for Zimbabwe 53, Mozambique 35, Nigeria 45, and Uganda 58. Select country‐specific results were additionally presented in two studies 19, 55. Modelling which applied to the region of SSA occurred in three studies 42, 43, 55, and there was one global modelling application 51.

### 3.1 Assessment of modelling studies

Thirty strategies of PrEP use were defined from the review of the modelling literature. Table 1 details the strategies of PrEP use identified, the number of relevant modelling studies which pertain to each strategy, the assessment of each strategy across each criterion, and the overall assessment for the strength of the available modelling evidence to inform decision making, for each strategy. No strategies were supported with modelling evidence that met all criteria to receive a green designation of quality, and no strategies failed across all criteria to receive a red designation. For all strategies, the available modelling evidence failed to meet one or more of the defined criteria and received an amber designation. Half of the strategies of PrEP use were supported with modelling evidence which used now‐outdated model parameters. There was only limited agreement in the findings across studies for each strategy. None of the studies which conducted cost‐effectiveness analyses complied with standards of reporting for economic evaluations, but nearly all studies met the rigour criteria for the quality of the modelling analyses, Table 1.

**Table 1:** Assessment of modelling evidence to inform strategies of PrEP implementation in sub‐Saharan Africa

PrEP strategies in sub‐Saharan Africa	Current	CEA compliance	Study agreement	Rigour	Traffic light assessment	Evidence for CE (0.5 × GDP)
PrEP for serodiscordant couples						
Short‐term PrEP for HIV‐ until HIV+ partner achieves viral suppression (4)	−	−	+	+	Amber	**Yes**
PrEP during conception & pregnancy for HIV‐ women in discordant partnerships (3)	−	−	−	+	Amber	**Yes**
Study‐setting delivery of ART and PrEP programme for high‐risk serodiscordant (2)	+	−	−	+	Amber	No
Government delivery of ART and PrEP programme for high‐risk serodiscordant (1)	+	−	−	+	Amber	No
PrEP for women						
PrEP for all women (7)	−	−	+	+	Amber	**Yes**
PrEP for adolescent girls and young women (9)	−	−	+	+	Amber	**Yes**
Short‐term PrEP for women during periods of high HIV risk (1)	−	−	−	+	Amber	No
PrEP for female sex workers (5)	−	−	+	+	Amber	No
PrEP for high‐risk female sex workers (1)	+	−	−	+	Amber	No
PrEP for “high risk” women (2)	+	−	+	−	Amber	No
PrEP for women and universal ART						
PrEP for adolescent girls and young women in context of universal ART (2)	+	−	+	+	Amber	No
PrEP for women in context of universal ART (1)	+	−	−	+	Amber	No
PrEP for female sex workers in context of universal ART (1)	−	−	−	+	Amber	No
PrEP for men						
PrEP for adolescent boys and young men (3)	−	−	−	+	Amber	No
Targeted outreach of MSM for combination prevention including PrEP (2)	−	−	−	+	Amber	No
PrEP for high‐risk MSM (1)	−	N/A	−	+	Amber	No
PrEP for male sex workers (1)	+	−	−	+	Amber	No
PrEP for “high risk” males (2)	+	−	+	−	Amber	No
PrEP use in the general population						
PrEP for the general population (12)	−	−	+	+	Amber	**Yes**
PrEP for the general population in context of universal ART (1)	+	−	−	+	Amber	**Yes**
PrEP for highly sexually active (6)	−	−	+	+	Amber	**Yes**
PrEP on demand (1)	+	N/A	−	+	Amber	No
PrEP for HIV‐ sexual partners of new HIV+ diagnoses, i.e. contact tracing (1)	+	N/A	−	+	Amber	No
Resource optimization, combination prevention and PrEP						
Optimization of HIV resources and combination prevention in sub‐Saharan Africa (2)	+	−	+	+	Amber	No
Optimization of HIV resources and combination prevention in South Africa (2)	−	−	+	+	Amber	No
Optimization of HIV resources and combination prevention in Kenya (3)	−	−	+	+	Amber	No
Optimization of HIV resources and combination prevention in Zambia (1)	−	−	−	+	Amber	No
Optimization of combination prevention for serodiscordant couples (1)	+	−	−	+	Amber	No
Optimization of fixed amount of antiretrovirals in the context of 90‐90‐90 & PrEP (1)	+	−	−	−	Amber	No
PrEP for populations where HIV incidence is >3 per 100 person‐years (1)	+	−	−	+	Amber	No

The number of modelling studies pertaining to each strategy is indicated in parentheses. “+” denotes all modelling analyses for a given strategy met the criteria, “−” denotes failure to meet criteria. The criteria assessment criteria are defined as follows: *Current:* Use of current modelling assumptions and scenarios. *CEA compliance:* Cost‐effectiveness analyses comply with the Gates Reference Case for economic evaluations 17. *Study agreement:* The presence of two studies with similar results. *Rigour:* Modelling analyses comply with criteria adapted from Garnett, *et al*. 18 recommended for use to evaluate the quality of modelling. Any evidence for cost‐effectiveness at the revised threshold of 0.5 times GDP per capita is denoted in bold for each strategy. ART, antiretroviral treatment; CE, cost‐effectiveness; CEA, cost‐effectiveness analysis; GDP, gross domestic product; HIV, human immunodeficiency virus; MSM, men‐who‐have‐sex‐with‐men; PrEP, pre‐exposure prophylaxis.

### 3.2 Cost‐effectiveness of PrEP with a revised threshold

When the cost‐effectiveness threshold was reduced to 0.5 times GDP per capita, PrEP was found unlikely to be cost‐effective in nearly all analyses (Table 1). There were six exceptions. Two analyses for South Africa, which use now outdated ART assumptions (CD4 eligibility <200 cells/μL), found PrEP for serodiscordant couples potentially cost‐saving 39, and PrEP for females cost‐effective 55. Alistar *et al*. 24 found PrEP for the general population in South Africa cost‐effective prior to universal treatment; however, when all HIV‐positive individuals were eligible for ART, PrEP remained cost‐effective only with low ART coverage. Similarly, Nichols *et al*. 46 find in their more recent analysis (in contrast to earlier findings 47) that PrEP for highly sexually active individuals in a rural setting in Zambia was no longer cost‐effective with the assumption of expanded ART eligibility criteria (CD4 <500 cells/μL). Nearly all countries in SSA have now adopted universal treatment of people living with HIV regardless of CD4 count as policy 64, and considerable ART scale‐up has already occurred 65.

In the remaining two studies, Walensky *et al*. 57 found PrEP for adolescent girls and young women in South Africa cost‐saving compared to a scenario of no PrEP. And Price *et al*. 49 find, in an analysis of PrEP during pregnancy and breastfeeding compared to no PrEP, that the potential beneficial impact of PrEP on reducing maternal and infant HIV infections outweighs even a substantial increase in the risk of pre‐term birth from PrEP drug exposure.

### 3.3 Summary of the modelling evidence for key priority populations

Serodiscordant couples, adolescent girls and young women, sex workers, and men who have sex with men are key priority populations for PrEP implementation in sub‐Saharan Africa. Below, we broadly summarize the available modelling evidence for the impact, cost and cost‐effectiveness of PrEP use among these populations. Table 2 details the specific findings from the modelling analyses.

**Table 2:** Impact, cost and cost‐effectiveness of oral PrEP strategies from modelling studies

PrEP strategies in sub‐Saharan Africa	Modelling	Location	Impact	Cost	Cost/infection averted	Cost‐effectiveness 1 or 3 × GDP	Cost‐effectiveness 0.5 × GDP
PrEP for serodiscordant couples							
Short‐term PrEP for HIV‐ until HIV+ partner achieves viral suppression	Mitchell 45	Nigeria	10% reduction in new infections (20 years)			**ICER $7870/DALY averted**	Not CE
	Jewell 35	South Africa				**$10,383/DALY averted**	Not CE
	Ying 58	Kampala, Uganda	*43% reduction in new infections (10 years, PrEP+ART)*	*$1058/couple‐years* ($408 for PrEP)		ICER $5354/DALY averted	Not CE
	Hallett 39	South Africa	10% to 49% reduction in new infections (age 50)		Cost‐saving‐$21,000	**Cost‐saving‐$4900/QALY saved**	**CS – borderline CE** a
PrEP during conception and pregnancy for HIV‐ women in discordant partnerships	Price 49	SSA	↓ in mother/infant HIV incidence outweighs possible ↑ in pre‐term birth			**ICER $965/DALY averted**	**CE**
	Hoffman 60	Non‐specific	0.1% ↑ in probability of successful outcome (when partner on ART)	$333/person‐years for PrEP			
	Hallett 39	South Africa	1% to 10% reduction in new HIV infections		Cost‐saving‐$12,000		
Study‐setting delivery of ART and PrEP programme for high risk serodiscordant	Ying 58	Kampala, Uganda	*43% reduction in new HIV infections (10 years, PrEP+ART)*	*$1058/couple‐years* ($408 for PrEP)		ICER $5354/DALY averted	Not CE
	Balzer 27	SSA	1.7% to 2% reduction in incidence (four years)				
Government delivery of ART and PrEP programme for high risk serodiscordant	Ying 58	Kampala, Uganda		*$453/couple‐years* ($92 for PrEP)			
PrEP for women							
PrEP for all women	van de Vijver 53	Zimbabwe	21% to 33% reduction in the risk of HIV infection				
	Pretorius 48	South Africa	5% to 12% of infections averted (10 years, PrEP for 15 to 35 F)		$12,500 to >$20,000 (10 years)		
	Abbas 2011 20	Non‐specific SSA	Up to 19% of infections averted (10 years)				
	Cremin 2013 36	KZN, South Africa	16% to 30% of infections averted (10 years, PrEP for 15 to 35 F)		$6000 to $6300 (10 years)		
	Verguet 55	42 SSA countries	557,000 infections averted (five years)			ICER $3800/DALY (five years)	Not defined
	Verguet 55	Nigeria	80,700 infections averted (five years)			ICER $4700/DALY (five years)	Not CE
	Verguet 55	Ethiopia	28,800 infections averted (five years)			ICER $8200/DALY (five years)	Not CE
	Verguet 55	DRC	13,600 infections averted (five years)			ICER $11,500/DALY (5 years)	Not CE
	Verguet 55	South Africa	119,800 infections averted (five years)			**ICER $700/DALY (five years)**	**CE**
	Verguet 55	Tanzania	34,600 infections averted (five years)			ICER $3000/DALY (five years)	Not CE
	Verguet 55	Kenya	35,300 infections averted (five years)			ICER $3000/DALY (five years)	Not CE
	Verguet 55	Sudan	8400 infections averted (five years)			ICER $13,000/DALY (five years)	Not CE
	Verguet 55	Uganda	31,800 infections averted (five years)			ICER $2200/DALY (five years)	Not CE
	Verguet 55	Ghana	6600 infections averted (five years)			ICER $10,600/DALY (five years)	Not CE
	Verguet 55	Mozambique	33,900 infections averted (five years)			**ICER $1200/DALY (five years)**	Not CE
	Verguet 55	Cote d'Ivoire	9400 infections averted (five years)			ICER $5400/DALY (five years)	Not CE
	Verguet 55	Cameroon	14,300 infections averted (five years)			**ICER $3400/DALY (five years)**	Not CE
	Verguet 55	Angola	5600 infections averted (five years)			**ICER $8600/DALY (five years)**	Not CE
	Verguet 55	Niger	1600 infections averted (five years)			ICER $23,600/DALY (five years)	Not CE
	Verguet 55	Burkina Faso	2900 infections averted (5 years)			ICER $ 16,800/DALY (five years)	Not CE
	Supervie 52	Botswana	39% of infections averted (10 years)				
	Blaizot 2016 30	Western Kenya	22% to 28% reduction in incidence (four years)				
PrEP for adolescent girls and young women	Abbas 2007 19	Non‐specific SSA	*Up to 46% reduction in cumulative new infections (10 years)*		*$5723 to $67,970*		
	Abbas 2011 20	Non‐specific SSA	*Up to 18% of infections averted (10 years)*				
	Cremin 2013 36	KZN, South Africa	*3.2% reduction in new infections* (10 years, PrEP for 7.3% of 15 to 24 years)	*Fixed $50 M/years*	*$10,540*		
	Blaizot 2016 30	Western Kenya	22% to 28% reduction in incidence (four years)				
	Walensky 57	South Africa	14% decline in new infections amongst AGYW (lifetime)	$5270/person; $217 M/years 1; $1.1 B 5 years	$10,100	ICER $/life‐years saved: **cost‐saving**	**CS**
	Alsallaq 25	Nyanza, Kenya	11,000 infections averted, 0.5 M DALYs (20 years, 6% 20 to 24 F)	Additional $31.8 M over 20 years			
	Blaizot 2017 29	KZN, South Africa	Additional 7% reduction in HIV incidence due to PrEP (10 years, 40% 15 to 24 F)				
	Chiu 32	South Africa	406,120 life‐years saved	Additional $10.7 B over 20 years		ICER (cost/LYS): $26,375	Not CE^a^
	Meyer‐Rath 44	South Africa	412,361 life‐years saved	Additional $10 B over 20 years		ICER (cost/LYS): $19,985	Not CE
Short‐term PrEP for women during periods of high HIV risk	Cremin 2015 35	Gaza, Mozambique	Approximately 5% to 49% of infections averted		$9538 (5y)		
PrEP for female sex workers	Vissers 56	Botswana	26 to 251 infections averted/100,000 person‐years (up to 29,400 infections)				
	Vissers 56	Nyanza, Kenya	44 to 342 infections averted/100,000 person‐years				
	Bekker 28	South Africa	Approximately 6% reduction in new infections amongst FSW (10 years)				
	Cremin 2017 33	Nairobi, Kenya			$65,160 ($43,520 to 90,250)		
	Chiu 32	South Africa	20,554 life‐years saved (20 years)	Additional $204 M over 20 years		**ICER (cost/LYS) $9947**	Not CE^a^
	Meyer‐Rath 44	South Africa	20,831 life‐years saved (20 years)	Additional $206 M over 20 years		**ICER (cost/LYS) $7473**	Not CE
PrEP for high risk female sex workers	Cremin 2017 33	Nairobi, Kenya	Small absolute impact due to small size of this population		$10,920 ($4700 to 51,560)		
PrEP for high risk women	Stover 51 Cremin 2015 34	Global Nyanza, Kenya	*16% to 19% reduction in new infections (40 years) approximately 11% of infections averted (10 years)*	*$20 M per year*	*$2060 to $36,350*	*Incremental cost/QALY $3500 to 3800* (40 years)	
PrEP for women + Universal ART							
PrEP for adolescent girls and young women in context of universal ART	Cremin 2013 36	KZN, South Africa	*41% infections averted* (10 years, 40% coverage 15 to 24 years +TasP)	$2.3 B (PrEP cost 10 years, $4.1 B total)	$39,900 (compared to TasP)		
	Alsallaq 25	Nyanza, Kenya	11,000 infections averted, 0.5 million DALYs (20 years, 6% 20 to 24 years)	Additional $31.8 M over 20 years			
PrEP for all women in context of universal ART	Cremin 2013 36	KZN, South Africa	*59% of infections averted (10 years, PrEP for 15 to 54 years +TasP)*	$7.7 B (10 years, PrEP cost; $9.5 B total)	$20,500		
PrEP for FSW in context of universal ART	Bekker 28	South Africa	*11% reduction in incidence among FSW* (*10 years, oral+topical PrEP +TasP)*				
PrEP for men							
PrEP for adolescent boys and young men	Cremin 2013 36	KZN, South Africa	*3.2% reduction in new inf* (*10 years, PrEP for 7.3% of 15 to 24 years)*	*Fixed $50 M/years (10 years)*	*$10,540 (10 years)*		
	Abbas 2007 19	Non‐specific SSA	*Up to 46% reduction in cumulative new infections (10 years)*		*$5723 to $67,970*		
	Abbas 2011 20	Non‐specific SSA	*Up to 18% reduction in cumulative new infections (10 years)*				
Targeted outreach of MSM for combination prevention including PrEP	Brookmeyer 31	South Africa	*1/3 reduction in new infections (five years)*				
	Cremin 2017 33	Nairobi, Kenya				PrEP enters optimal portfolio at high budgets	
PrEP for high‐risk MSM	Brookmeyer 31	South Africa	10% to 12% of infections averted (five years)				
PrEP for male sex workers	Cremin 2017 33	Nairobi, Kenya				PrEP enters optimal portfolio at similar budget levels to earlier ART (CD4<350)	
PrEP for high risk men	Stover 51 Cremin 2015 34	Global Nyanza, Kenya	*16% to 19% reduction in new infections* (40 years) *approximately 11% infections averted* (10 years)	*$20 M per year*	*$2060 to $36,350*	*Incremental cost/QALY $3500 to 3800* (40 years)	
PrEP use in the general population							
PrEP for the general population	Abbas 2007 19	Non‐specific SSA	Up to 74% reduction in cumulative new infections (PrEP for 25% to 75%, 10 years)		$6812 to $67,842		
	Abbas 2011 20	Non‐specific SSA	Up to 40% reduction in cumulative new infections (PrEP for 15% to 60%, 10 years)				
	Supervie 52	Botswana	Up to 40% reduction in new infections (10 years)				
	Dimitrov 37	Non‐specific SSA	53% to 61% of infections averted (PrEP for 60%, 10 years; 20 years)				
	Abbas 2013 21	South Africa	21% of infections averted (PrEP for 30%, 10 years)				
	Verguet 55	42 SSA countries	390,000 infections & 5.4 M DALYs averted (PrEP for 10%, five years)^b^	$36 B (five years)^b^		ICER $5800/DALY (five years)^b^	Not defined
	Long 41	South Africa	28% of infections averted (PrEP for 50%, 10 years)	$84 B (10 years)		**ICER $9000/QALY (10 years)**	Not CE^a^
	Cremin 2013 36	KZN, South Africa	3.6% infections averted (PrEP for 4.4%, 10 years)	$50 M/yr (10 years)	$9390		
	Zhao 59	South Africa	24 years to achieve 50% reduction in incidence (PrEP for 20%)				
	Alistar 24	South Africa	*Up to 63% of infections averted (3.3 to 3.8 M); 990 M‐1 B QALYs gained (20 years)*	*$335 to 351 B (20 years)*		**$1600 to 2650/QALY gained**	**CE**
	Nichols 2013 47	Macha, Zambia	23% reduction in new infections; 23,571 QALYs gained (10 years)	$43.9 M ($41.4 to 46 M, 10 years)			
	Nichols 2014	Macha, Zambia	59% reduction in new infections (PrEP +ART<500, 40 years)	$173.6 M (40 years)		ICER $5861/QALY gained	Not CE
PrEP for the general population in context of universal ART	Alistar 24	South Africa	4 to 4.6 M infections averted, 1 B QALYs gained (20 years)	$351 to 366 B (20 years)		**$4300 to 13,300/QALY gained (20 years)**	**CE at low ART coverage**
PrEP for highly sexually active	Abbas 2007 19	Non‐specific SSA	Up to 29% reduction in cumulative new infections (10 years)		$638 to $9923		
	Abbas 2007 19	Southern SSA	2.7 to 3.2 M infections averted (10 years)				
	Abbas 2007 19	Lesotho	92,710 infections averted (10 years)				
	Abbas 2007 19	Botswana	132,870 infections averted (10 years)				
	Abbas 2007 19	Zambia	361,132 infections averted (10 years)				
	Abbas 2007 19	South Africa	1.5 M infections averted (10 years)				
	Abbas 2011 20	Non‐specific SSA	Up to 8% reduction in cumulative new infections (10 years)				
	Nichols 2013 47	Macha, Zambia	1/3 reduction in new infections; 32,216 QALYs gained (10 years)	$11.5 M ($11.1 to 13.4 M, 10 years)		**$323/QALY gained (10 years)**	**CE**
	Nichols 2014 46	Macha, Zambia	16% to 45% reduction in new infections (40 years)			Not CE (results not given)	
	Alistar 24	South Africa	1.8 to 3.1 M infections averted, 962 to 978 M QALYs (20 years)	$275 to 277 B (20 years)			
	Balzer 27	Non‐specific SSA	1.4% reduction in incidence (four years)				
PrEP on demand	Balzer 27	Non‐specific SSA	0.6% reduction in incidence (four years)				
PrEP for HIV‐ sexual partners of new HIV+ diagnoses (i.e. contact tracing)	Balzer 27	Non‐specific SSA	Negligible PrEP effect (four years)				

Bold indicates cost‐effective at defined threshold. Outcomes in italics indicates combined PrEP impact/cost/cost per infection averted – across multiple interventions or across multiple populations. AGYW, adolescent girls and young women; ART, antiretroviral treatment; B, billion; CE, cost‐effectiveness; CS, cost‐saving; DALY, disability adjusted life year; FSW, female sex worker; GDP, gross domestic product; HIV, human immunodeficiency virus; ICER, incremental cost‐effectiveness ratio; KZN, KwaZulu‐Natal; LYS, life‐year saved; M, million; MC, male circumcision; MSM, men who have sex with men; MSW, male sex worker; PrEP, pre‐exposure prophylaxis; QALY, quality adjusted life year; SSA, sub‐Saharan Africa; TasP, treatment as prevention.

^a^CE threshold was not defined in analysis, designation based on relevant approximate GDP per capita for year of analysis; ^b^impact, cost, CE results are available for each of the 42 countries modelled.

#### 3.3.1 Serodiscordant couples

Two modelling studies investigated the impact in South Africa of short‐term PrEP for the HIV‐negative partner, prior to viral suppression in the HIV‐positive partner, and found this was a cost‐effective 35, and potentially cost‐saving 39, intervention in this setting. In Kampala, Uganda, an intervention of PrEP and ART targeted to 90% of high‐risk serodiscordant couples just exceeded the cost‐effectiveness threshold, but the timeframe of this analysis (10 years) may have been insufficient to fully capture the effects of PrEP on DALYs averted 58. In an optimization analysis of combination prevention for serodiscordant couples in Nigeria, it was cost‐effective to first prioritize ART access for all HIV‐positive partners and condom promotion together with implementing short‐term PrEP for the HIV‐negative partner 45. The impact of PrEP across these studies ranged from a 10% to 49% reduction in new infections. Ying *et al*. 58 conducted a micro‐costing study of real‐world delivery of a PrEP and ART programme for serodiscordant couples in a demonstration project conducted in Kampala, Uganda, and then estimated the cost of the programme if provided by the government. In the research study setting, PrEP and ART for discordant couples cost $1058 per couple‐year, with the PrEP component accounting for $408 of this cost. It was estimated that when implemented in public clinics, the same programme would cost $453 per couple‐year, with PrEP accounting for $92 of this cost. The assumptions used for the annual cost of PrEP in the studies in Nigeria and South Africa fall near the midpoint of the Ying *et al*. estimates, with a PrEP cost of $233 per patient‐year assumed in the Nigeria study 45, and $200 (range $150 to 250) 39 and $250 per patient‐year 35 assumed in the South African studies. Additional File S2 details the assumed costs for PrEP and ART across studies.

#### 3.3.2 Adolescent girls and young women

Donors and global actors, notably the US President's Emergency Plan for AIDS Relief (PEPFAR) are focused on adolescent girls and young women as a key priority population for PrEP implementation in sub‐Saharan Africa. Walensky *et al*. 57 estimate that PrEP for high‐risk adolescent girls and young women in South Africa can be a cost‐saving intervention (compared to no PrEP) and can result in a 14% decline in new HIV infections. The total estimated cost of this strategy in South Africa is $1.1 billion over five years with a cost per infection averted of $10,100 57. Cremin *et al*. 36 report a similar cost per infection averted ($10,540) providing PrEP to adolescents and youth (both sexes) in Kwa‐Zulu Natal, but find the intervention would not be cost‐saving unless the cost of PrEP was reduced to a fraction (1/6th) of the estimated cost per person‐year. In a non‐specific generalized epidemic setting, Abbas *et al*. 20 estimate that up to 18% of new infections can be averted with a PrEP strategy targeted by age (but not by gender). These findings are more conservative than previous estimates from the author of up to 46% of new HIV infections averted at a cost per infection averted ranging from $5723 to $67,970 19. Alsallaq *et al*. 25 find that in Nyanza, Kenya a youth‐focused strategy of combination prevention which includes PrEP for high‐risk adolescent girls and young women can be cost‐effective.

#### 3.3.3 Sex workers

Modelling of PrEP for sex workers illustrates the overall effect of PrEP use by sex workers reduces as HIV epidemics become generalized (due to the resultant decreased contribution of sex work to the overall epidemic in the models) and with high rates of condom use by sex workers 28, 40. PrEP is predicted to be able to reduce HIV risk even if it results in reductions in condom use 38, but increased condom use is predicted to have a similar or greater impact than PrEP 28, 56, 61. Analyses which aim to optimize a fixed amount of HIV prevention resources in Nairobi, Kenya found that PrEP for male sex workers enters the optimal intervention portfolio at similar levels of spending as ART, but very high budget availability is needed before PrEP for female sex workers enters the optimal portfolio indicating a low probability of cost‐effectiveness 33. Prioritizing PrEP for the sex workers at greatest risk can improve cost‐effectiveness. Cremin *et al*. 33 estimate the cost per infection averted can be reduced from $65,160 to $10,920 with prioritization of PrEP to female sex workers at highest risk in Nairobi. However, the absolute impact of this strategy is minor as a result of the small size of the targeted population, a finding that applies to other highly focused PrEP strategies 35.

#### 3.3.4 Men who have sex with men

Among MSM, Brookmeyer *et al*. 31 illustrate 0% to 12% of new HIV infections can be averted with prioritized PrEP for high‐risk MSM in South Africa. Cremin *et al*. 33 find that in Nairobi, Kenya, PrEP for MSM enters an optimal combination prevention portfolio at very high budget availability ($175 million over 10 years). Additional studies 26, 42, 43, 51 have modelled the implementation of PrEP amongst MSM but do not present specific results for this priority population.

### 3.4 Summary of the role of PrEP in combination prevention and with ART scale‐up

A greater impact was observed with combination prevention portfolios that include PrEP compared to those that did not 25, 30, 36, 51. Modelling indicates that PrEP has an increasing role in optimal prevention portfolios at high budget availability 42, but other interventions, including enhanced testing and ART, male circumcision and condom promotion, may be more cost‐effective for prioritization 25, 33, 45, Table 3. A single analysis investigated resource optimization and the use of a specific incidence benchmark for PrEP eligibility. The threshold under investigation was PrEP for populations where HIV incidence is greater than three per 100 person‐years, corresponding with the suggested threshold for PrEP eligibility from the World Health Organization 66. This analysis 42 found that at low budget availability, there can be a loss of impact with the use of threshold PrEP, while at high budget availability threshold PrEP limits the potential impact of PrEP. This is because when resources are scarce, threshold PrEP draws resources away from other more cost‐effective interventions. However, with greater resource availability a greater impact may be achieved by additionally providing PrEP to populations which fall below the requisite incidence threshold.

**Table 3:** PrEP in the context of resource optimization and combination prevention

PrEP strategies in sub‐Saharan Africa	Modelling	Location	Impact	Cost	Cost‐effectiveness
Resource optimization, combination prevention and PrEP					
Optimization of HIV resources and combination prevention in SSA	McGillen 43	SSA	3×impact targeting by risk + geo; greater marginal impact by risk versus geo		
	McGillen 42	SSA	Marginal impact of PrEP at high budget availability	$6 B+ (15 years)	Key priorities: MC, behavioural change communication for high risk, early ART
	McGillen 42	SSA	14% reduction in new infections (15 years)	$1T (15 years)	
Optimization of HIV resources and combination prevention in South Africa	Smith 50	South Africa			Optimal: MC, ART for all with outreach testing
	Long 41	South Africa	*62% infections averted, 31 M QALYs (testing+ART+MC+microbicides+PrEP)*		$10,000 to 30,000/QALY gained
Optimization of HIV resources and combination prevention in Kenya	Anderson 26	Kenya	Optimal: 40% reduction in new infections, +14% targeting by risk + geo	$600 M (15 years)	Optimal (uniform): Behavioural change communication, early ART, then PrEP
	Alsallaq 25	Nyanza, Kenya	11,000 infections averted, 0.5 M DALYs (20 years)	$31.8 M (20 years)	Optimal (youth): HIV testing, TasP, condoms, MC, PrEP
	Cremin 33	Nairobi, Kenya			Optimal: Condom promotion for MSM & MSW, ART retention, earlier ART, MC, then PrEP
Optimization of HIV resources and combination prevention in Zambia	Nichols 46	Macha, Zambia			Optimal: ART expansion. PrEP economical only at very high budget availability
Optimization of combination prevention for serodiscordant	Mitchell 45	Nigeria			Optimal: ART scale‐up, condoms, TasP, then PrEP
Optimization of fixed amount of antiretrovirals in the context of 90‐90‐90 and PrEP	Akudibillah 23	South Africa			Optimal: Use of all antiretrovirals for ART
PrEP for populations where HIV incidence is >3 per 100 person‐years	McGillen 42	SSA	Loss of impact	Low budget	At low budgets threshold PrEP takes away from more cost‐effective interventions
	McGillen 42	SSA	Reduced impact: 7% of incidence reduction lost compared to optimization	High budget	At higher budgets threshold PrEP limits optimal impact to lower incidence pops

Impact in italics indicates combined PrEP impact across multiple interventions. ART, antiretroviral treatment; B, billion; geo, geography; HIV, human immunodeficiency virus; M, million; MC, male circumcision; MSM, men who have sex with men; MSW, male sex worker; PrEP, pre‐exposure prophylaxis; QALY, quality adjusted life year; SSA, sub‐Saharan Africa; T, trillion; TasP, treatment as prevention.

The combined effect of PrEP and ART is of great interest given the successful scale‐up of ART. While ART results in the greatest singular impact, PrEP and ART in combination lead to greater reductions in incidence than either intervention alone 22, 30, 54, 58. The magnitude of the impact of PrEP will depend on ART use with a greater PrEP effect observed at lower ART coverage 27. In generalized epidemic settings, the effect of PrEP at the population level reduces as eligibility and use of ART expands. Correspondingly, the cost‐effectiveness of PrEP decreases as ART coverage increases. Both Pretorius *et al*. 48 and Alistar *et al*. 67 find that PrEP for the general population does not provide value for money in the context of ART scale‐up. The general consensus from optimization analyses is that it is better value to prioritize expanding ART access before adding PrEP 25, 33, 36, 41, 46, 50, Table 3. When there is very high budget availability, PrEP can be a cost‐effective addition 42, 46; however, the likelihood of such high budgets is unknown.

### 3.5 Gaps in the modelling of oral PrEP

Several key gaps in the modelling of oral PrEP were identified during this review, Box 1. The assessment of the evidence highlighted the modelling literature available to inform policy and decision making is now outdated. This includes now outdated assumptions for key model parameters – PrEP efficacy and effectiveness, for example – but importantly it includes the intervention assumptions made in both the baseline status quo scenarios and in the intervention scale‐up and comparison scenarios. This is mainly due to recent changes in ART eligibility and the substantial expansion of testing and treatment which occurred after many of the analyses were conducted. The modelling of oral PrEP interventions has often used what are now considered optimistic assumptions for PrEP uptake, adherence and retention. These assumptions were made in the absence of available data and without the incorporation of additional activities designed to increase uptake, adherence or retention. As a result, there remains a limited understanding of the potential impact of programme delivery when data from PrEP studies or programmes are used in place of model assumptions. These factors remain an important consideration for each priority population.

Box 1 **Gaps in the modelling of PrEP and recommendations for data collection and future modelling**

1 Gaps identified in the modelling of oral PrEP in sub‐Saharan Africa:

- Model assumptions, scenarios and data inputs are now outdated for many modelling analyses
- Limited modelling (impact, cost, cost‐effectiveness) of:
  – PrEP strategies adopted in SSA
  – PrEP amongst adolescent girls and young women
  – PrEP amongst male populations
- Lack of cost of real‐world programme implementation by priority population, including outreach, linkage and retention, health system constraints and potential economies of scope
- Economic evaluations tend not to follow standards of reporting
- Neglect of other benefits, for example the benefit of PrEP programmes including linkages to testing, treatment, and sexual and reproductive health services

Priority areas for data collection to inform modelling of oral PrEP:

- Real‐world programme costs, including outreach for priority populations
- Uptake of PrEP disaggregated by priority population, and costs of improvement
- Adherence to PrEP disaggregated by priority population, and costs of improvement
- Preferences for PrEP disaggregated by priority population
- Retention in PrEP programmes disaggregated by priority population
- Outcomes of patients disengaged from PrEP programmes

Recommendations for further modelling of oral PrEP:

- Modelling the impact and cost‐effectiveness of PrEP for priority populations based on the actual programmes that are being implemented and using data from studies for uptake, adherence and retention
- Representation of the full costs of programme delivery by priority population, including cost of outreach and engagement
- Modelling which investigates the potential additional benefits of PrEP programmes, including linkage to testing, ART, sexual and reproductive health services
- Inclusion of economies of scope, health system constraints, equity and equity‐efficiency trade‐offs
- Modelling the impact, cost and cost‐effectiveness of the PrEP strategies adopted and implemented in sub‐Saharan Africa
- Economic evaluations that follow standards of reporting

The modelling of PrEP in SSA has given a greater focus to female priority populations compared to male populations. Adolescent girls and young women are the key focus of PEPFAR's Determined, Resilient, Empowered, AIDS‐free, Mentored and Safe women (DREAMS) programme, and in many PrEP demonstration and implementation projects and national programmes, but the modelling available has yet to incorporate the uptake and adherence observed in studies to quantify the potential impact of PrEP use in this population. Among men, there has been limited modelling of PrEP for men at potentially higher‐risk of acquiring HIV in SSA, including PrEP for MSM, male sex workers and the male clients of female sex workers.

The incorporation of supply‐side constraints remains another gap in the modelling. The intervention scenarios used to model PrEP assume that the existing human resources will be able to cope with the introduction and scale‐up of PrEP programmes. This assumption may not be realistic and is perhaps inappropriate in studies with assumptions for high intervention coverage. While supply‐side constraints would be expected to limit the impact of PrEP, other factors may increase its impact. For example, it is possible there are additional benefits of PrEP programmes that have not been quantified. The linkages from PrEP programmes to HIV testing and treatment provision for those HIV positive, or from access to the sexual and reproductive health services which often accompany PrEP programmes, for example diagnosis and treatment of sexually transmitted infections, could result in reductions in HIV incidence. These potential additional benefits have yet to be directly explored in the modelling.

In terms of costs and cost‐effectiveness, there will likely be specificities of delivering PrEP to different populations which will impact the associated costs of targeting and recruitment. For example, the potential for increased costs to find and reach female sex workers or MSM. The inclusion of the costs to locate and engage the priority populations targeted by PrEP programmes remains a key gap in the modelling analyses conducted. There may also be potential for economies of scope. For example, recruitment of priority populations through sexual and reproductive health, maternal and child health facilities or through schools.

There has been limited inclusion of costing from primary data collection in the cost‐effectiveness analyses included. Notable exceptions include Ying *et al*. 58, Eakle *et al*. 68 and Cremin *et al*. 33. Modelling analyses to date have also assumed PrEP integration into ongoing services, or the establishment of standalone PrEP services, can be done within the scope of the current resources available in already strained health systems. These assumptions may not be realistic.

Standards of reporting for economic evaluations have been produced with the aim to better inform decision making in health. None of the cost‐effectiveness analyses in this review were compliant with the new Gates Reference Case for economic evaluations 17. Specifically, full programme costs were generally not included, there was limited inclusion of the estimated change in costs with intervention scale‐up, the use of DALYS to report cost‐effectiveness was not universal, many studies did not include discounting (of costs and/or impact), and others used a time horizon that was too short to capture the full intervention effects 30, 31, 36, 41, 47, 48, 55, 56, 58. In a recent example from a high‐income setting, an analysis of the cost‐effectiveness of PrEP for MSM in the UK illustrates the sensitivity of the results with the use of different time horizons. With a 20‐year analysis, PrEP for MSM was not a cost‐effective strategy in this setting, but was cost‐effective with a 40‐year analysis, and was cost saving with an 80‐year analysis 69. Despite these gaps, countries in sub‐Saharan Africa have moved forward with adopting national PrEP policies and implementation is currently underway. These policies indicate broad eligibility criteria for PrEP in many settings 70. There is currently limited modelling in the published literature of the potential impact, cost and cost‐effectiveness at scale of the national PrEP strategies adopted in sub‐Saharan Africa.

### 3.6 Recommendations for future modelling and data collection

Previous *ex ante* modelling of oral PrEP helped the global community and country level policy makers assess the potential impact and cost effectiveness of this potential new technology. With the standard of care changing rapidly in countries and decisions being made to introduce PrEP, modelling at the country level can inform strategic (should PrEP be given to certain populations and not others?), and tactical decisions (should PrEP be provided in the community through mobile teams or in schools or facility‐based integrated into current sexual and reproductive health or maternal and child health services?). This new purpose of modelling will guide the definition of the comparison scenarios, data and the assumptions needed. To date, most modelling assumptions about the performance of the comparison scenarios for strategic decisions are now outdated. In future scenarios, assumptions will need to be made about further changes in the performance of these comparison scenarios, for example switching to dolutegravir for treatment will change the cost implications of treatment and the need for second line treatment and therefore the incremental cost savings that can be realized with PrEP.

Using models in the future for tactical decision making will require better local data on the costs and information on the outreach and engagement activities across populations. While the costs of service delivery of PrEP may be less variable for a given platform, the costs of outreach and engagement of populations will likely be a main driver of differences in PrEP delivery costs across populations. Future modelling should include costing data reflecting the specificities of delivering PrEP to different populations, and these costed activities should reflect the modelled effect in terms of increase in coverage. This is particularly important if the comparison is between the populations to prioritize. For example, PrEP delivery to women in serodiscordant couples through sexual and reproductive health or maternal and child health has the potential to realize economies of scope with less outreach needed. For sex workers, outreach and engagement are likely essential and will be the main driver of costs in tailored PrEP programmes for this population. In adolescent girls and young women, PrEP delivery may be done through schools, but there is a need for information campaigns that have not yet been costed.

The cost effectiveness of PrEP programmes will be affected by health system constraints. Future modelling could include these constraints either by limiting the feasible coverage of interventions within a realistic estimation of current capacity for expansion in a programme, estimating and accounting for the costs of relaxing health system constraints in the analysis (e.g. recruiting new nurses to expand the service), or varying the willingness to pay threshold depending on the constraints faced 71, 72.

Improved assumptions describing intervention effects will be essential for PrEP modelling to more directly inform policy decision making. Priority areas for data collection include PrEP uptake and adherence, retention in care and the outcomes of those disengaged from care for different priority populations, Box 1. Any activities to increase these factors to achieve modelled levels of coverage, programme performance and individual participation should also be incorporated. These data may soon be available from ongoing pilot and demonstration projects and will be necessary for future modelling to capture the real‐world costs to implement PrEP programmes.

Finally, models to date have informed decisions on whether to implement PrEP and which populations to prioritize based on value for money. Efficiency of investments is a key criterium for priority setting but may not be the only one. Future modelling can also inform policy makers interested in equity – that is having a broader commitment to secure a sufficient level of health for all and to narrow unjust inequalities 73 – by quantifying the possible equity efficiency trade‐offs that may be needed when prioritizing populations based on criteria other than cost‐effectiveness 74.

### 3.7 Limitations

The focus of this scoping review is on peer‐reviewed, published literature. It is recognized there will be additional unpublished modelling of oral PrEP (conference abstracts, grey literature); however, the peer‐review process was valued as an additional quality control mechanism. A single database that is broadly accessible to policy makers was used for the search. While cross‐referencing of the citations in the records retrieved with those returned in the PubMed search did not identify additional articles, it is possible that expanding the search to additional databases may uncover additional articles. The search strategy was intentionally broad to capture different types of modelling analyses; however, studies which quantify the cost of PrEP programmes but do not incorporate modelling did not meet our inclusion criteria. We did not identify any articles with relevance to SSA that were excluded on this basis, but these types of studies may not be captured with our search criteria which was focused on modelling of PrEP. It was not possible for the literature search, data extraction and assessment of evidence to be conducted in duplicate. Both co‐authors reviewed all results and resolved any queries or discrepancies that arose, but this remains a limitation.

The revised cost‐effectiveness threshold of 0.5 times GDP has been demonstrated as a more realistic threshold when the opportunity cost of healthcare spending is considered. However, there is limited available evidence to estimate opportunity costs across health, and further uncertainty regarding opportunity costs for HIV. There is also recent evidence which suggests this revised threshold may still be idealistic. A resource optimization analysis which used the committed HIV budget in South Africa to define a country‐specific cost‐effectiveness threshold for HIV prevention and treatment interventions, found the cost‐effectiveness threshold based on affordability was only a small fraction of GDP per capita 44.

## 4 Conclusions

The modelling of oral PrEP began when clinical efficacy trials were still underway and has now spanned more than a decade. The available modelling evidence base is now outdated and has yet to benefit from recent data collection in PrEP demonstration and implementation studies. The available evidence indicates that at traditional cost‐effectiveness thresholds PrEP can be a cost‐effective intervention in SSA, and this is a finding that has been widely reported. When a more representative cost‐effectiveness threshold is considered, there is limited evidence for the cost‐effectiveness of PrEP. This suggests PrEP may not currently be affordable in most settings in the region and it may be more cost‐effective to focus resources on expanding access to other interventions, notably ART, before adding PrEP. Updated modelling which captures the real‐world delivery of PrEP, uses current intervention scenarios and is parameterized with data from demonstration and implementation projects, is needed in support of more conclusive findings and actionable recommendations for programmes and policy.

The available modelling of PrEP has focused on strategies of use among key priority populations. It is not clear if prioritized PrEP is a feasible or acceptable policy at the national or programme level. From a policy perspective, issues surrounding programme implementation are often paramount for decision making. The prioritization of PrEP to specific populations, or to a specific sub‐set of higher risk individuals within a priority population, may present additional challenges for programmes. Challenges in finding and accessing these individuals, encouraging uptake of PrEP, and supporting retention in care, all of which may not be feasible or practical in over‐burdened health systems with resource constraints. Strategies of prioritized PrEP may also create stigma – stigma surrounding the use of PrEP itself, and stigma for the populations identified as eligible for PrEP and thus at‐risk for HIV. Furthermore, PrEP for priority populations runs counter to considerations of equity and the equitable access to the provision of HIV prevention options for those who self‐identify as in‐need, or desire to use PrEP. These are potentially important considerations from the policy perspective. More nuanced modelling approaches which combine the practicalities of programme implementation, incorporate supply and demand‐side constraints, and consider issues such as equity and potential equity‐efficiency trade‐offs are needed for the development of policy relevant PrEP strategies.

Future modelling applications should address these issues, at the same time aligning with countries to support the needs of programme planners and decision makers for models to more directly, and usefully, inform policy decisions and programme implementation.

## Competing interests

KKC has received personal fees from Imperial College London Consultants during the conduct of the study, and personal fees from Imperial College London Consultants and UNAIDS outside the submitted work. TBH reports personal fees from Imperial College London Consultants and the Bill and Melinda Gates Foundation outside the submitted work. GBG has received funds from BMGF for work outside the submitted work and was previously a co‐investigator for a PrEP demonstration project in South Africa.

## Authors’ contributions

KKC and TBH designed the study. KKC conducted the review, extracted the data and wrote the initial draft. TBH and GBG reviewed all results and contributed to writing the manuscript. All authors (KKC, TBH and GBG) reviewed and approved the final version.

## Abbreviations

AGYW: adolescent girls and young women
ART: antiretroviral treatment
CE: cost‐effectiveness
CEA: cost‐effectiveness analysis
DALY: disability adjusted life year
DREAMs: Determined, Resilient, Empowered, AIDS‐free, Mentored and Safe women
FSW: female sex workers
GDP: gross domestic product
ICER: incremental cost‐effectiveness ratio
LMIC: low and middle‐income countries
LYS: life‐years saved
MSM: men who have sex with men
HIV: human immunodeficiency virus
PEPFAR: US President's Emergency Plan for AIDS Relief
PrEP: pre‐exposure prophylaxis
QALY: quality‐adjusted life year
SSA: sub‐Saharan Africa

## Supporting information


**Additional File S1.** PRISMA‐ScR checklist.

**Additional File S2.** Supplementary Information.
