# Supplementary material for: The impact, cost and cost‐effectiveness of oral pre‐exposure prophylaxis in sub‐Saharan Africa: a scoping review of modelling contributions and way forward
Source: J Int AIDS Soc. 2019 Sep 19;22(9):e25390. doi: 10.1002/jia2.25390 (PMC6753289; doi:10.1002/jia2.25390)
Supplement: Supplementary file 2 — Additional File S2. Supplementary Information. [file JIA2-22-e25390-s002.docx]

**Additional File S2. Supplementary Information**

1. **Search Strategy**

Broad search criteria were used consisting of permutations of the following terms:

“modelling” AND “PrEP” AND “HIV”

The following is the explicit search string used in PubMed:

((((modelling[Title/Abstract] OR model[Title/Abstract])

AND

PrEP[All Fields]) OR ("pre-exposure prophylaxis"[MeSH Terms] OR ("pre-exposure"[All Fields] AND "prophylaxis"[All Fields]) OR "pre-exposure prophylaxis"[All Fields] OR ("pre"[All Fields] AND "exposure"[All Fields] AND "prophylaxis"[All Fields]) OR "pre exposure prophylaxis"[All Fields])) OR (preexposure[All Fields] AND ("prevention and control"[Subheading] OR ("prevention"[All Fields] AND "control"[All Fields]) OR "prevention and control"[All Fields] OR "prophylaxis"[All Fields])))

AND

("hiv"[MeSH Terms] OR "hiv"[All Fields])

AND

English[lang]

Only articles published in English were included. The initial search date was 5 June 2017, updated 30 Nov 2017. No restrictions were placed on publication date and all published results were included up to the final search 30 Nov 2017. The bibliographies of the papers retrieved, and all review papers, were screened for additional sources. Additional papers were identified through expert consultation. The first author conducted all aspects of the search and screened all records retrieved. Both co-authors reviewed all results for consistency and resolved any discrepancies or queries that arose during the search.

1. **Assessment Criteria**

**Assessment of quality and relevance of modelling evidence: Criteria defined for *rigour***

The following criteria for inclusion in modelling analyses were adopted from Garnett, et al, *Mathematical models in the evaluation of health programmes* [1]:

- 1. Discussion of model structure
     - *Appropriate detail to understand how analyses conducted including representation of natural history of disease and intervention effect*
  2. List of key model parameters
     - *Justification and/or rationale for values used (with citation as appropriate)*
  3. Assessment of model predictions with data
     - *Representation of model fit to data, goodness of fit, or out-of-sample prediction*
  4. Incorporation of uncertainty and/or sensitivity analyses
  5. Discussion and conclusions from modelling anlayses clearly supported by the model results

These indicators of model quality do not necessarily reflect on the accuracy of the results. However, they do help the reader assess the appropriateness of the approach used. All of the above criteria need to be met to receive a (+) designation in Table 1 of the main manuscript.

**Assessment of quality and relevance of modelling evidence: *CEA compliance***

The following criteria were evaluated for each cost-effectiveness analysis, derived from the recommended guidance on the use of evidence from economic evaluation to inform decision making in health in *The Gates Reference Case* [2]:

1. Clear communication and transparency
   - *Decision problem fully described*
   - *Limitations characterised*
   - *Declaration of interests reported*
2. Comparator(s)
   - *Current practice used as base case comparator*
3. Evidence
   - *Use or relevant evidence available for the decision problem*
4. Measure of health outcome
   - *Use of DALYs (QALYs can be used in separate analysis)*
5. Costs
   - *Costs reflective of intervention setting and target population(s)*
   - *Cost all resource implications relevant to the decision problem*
   - *Estimation of changes in costs due to scalability*
6. Time horizon and discount rate
   - *Lifetime time horizon (or sufficient to capture full costs and effects)*
   - *3% discount of costs and effects*
7. Heterogeneity
   - *Exploration of effect in different subpopulations*
8. Uncertainty
   - *Uncertainty arising from model/analysis structure and from parameters*
   - *Sensitivity analyses informing the implication of different estimates for key parameters*
9. Budget impact
   - *Estimate of impact on budget(s) where the intervention will be used*

Cost-effectiveness analyses were required to adhere to the above guidance in order to receive a (+) designation in Table 1 of the main manuscript.

The first author (KKC) conducted the assessment of the evidence. All results were reviewed by both co-authors (TBH, GBG). Any queries or discrepancies arising from the assessment were resolved by the co-authors (TBH, GBG).

1. **Assumptions made for PrEP and ART costs across studies**

**Range of PrEP and ART costs across studies:**

**Study-specific assumptions for PrEP and ART costs:**

| **Study** | **PrEP cost** | **ART cost** | **Location** | **Notes** |
| --- | --- | --- | --- | --- |
| Alistar [3] | $80 | $150 | South Africa |  |
| Walensky [4] | $150 | $192 | South Africa | *PrEP: drugs, chemistry panels, clinic visits; ART: 1st line ($412 for 2nd line)* |
| Pretorius [5] | $150 | $600 | South Africa |  |
| Smith JA [6] | $170-190 | $255-295 | South Africa |  |
| Hallett [7] | $150-250 | $450-800 | South Africa | *PrEP: lab testing, human resources, antiretrovirals* |
| Jewell [8] | $250 | $515 | South Africa | *Full PrEP costs* |
| Cremin [9] | $250 | $600 | South Africa | *ART: "total delivery cost"; PrEP: testing, HR, facilities, antiretrovirals* |
| Long [10] | $800 | $800 | South Africa | *ART: 1st line ($1200 for 2nd line)* |
| Chiu [11] | $219-285 | $317 | South Africa | *Costs converted from ZAR to USD using 31 Dec 2014 rate* |
| Alsallaq [12] | $200 | $168 | Kenya |  |
| Cremin [13] | $250 | $252 | Kenya |  |
| Anderson [14] | $250 | $515 | Kenya |  |
| Cremin [15] | $391-496 | $252 | Kenya | *Full PrEP costs* |
| Nichols [16] | $126 | $194 | Zambia |  |
| Nichols [17] | $134 | $221-230 | Zambia |  |
| Cremin [18] | $300 | $294 | Mozambique | |
| Mitchell [19] | $233 | $365 | Nigeria |  |
| Ying [20] | $92 | $361 | Uganda | *Adjustment of study-setting estimates to reflect delivery in government clinic* |
| Ying [20] | $408 | $650 | Uganda | *Micro costing - study setting* |
| Stover [21] | $95 | $515 | Global |  |
| McGillen [22] | $95 | $457 | Regional - SSA | |
| McGillen [23] | $95 | $457 | Regional - SSA | |
| Verguet [24] | $200 | $880 | Regional - SSA | |
| Abbas [25] | $208-700 | no ART | SSA setting | *Drug costs* |
| Price [26] | $315 | no ART | SSA setting |  |

**REFERENCES**

1. Garnett, G.P., et al., *Mathematical models in the evaluation of health programmes.* Lancet, 2011. **378**(9790): p. 515-25.

2. Bill and Melinda Gates Foundation, et al., *Bill and Melinda Gates Foundation Methods for Economic Evaluation Project (MEEP): Final Report*. 2014, National Institute for Health and Clinical Excellence International: London. p. 68.

3. Alistar, S.S., P.M. Grant, and E. Bendavid, *Comparative effectiveness and cost-effectiveness of antiretroviral therapy and pre-exposure prophylaxis for HIV prevention in South Africa.* BMC Med, 2014. **12**: p. 46.

4. Walensky, R.P., et al., *Potential Clinical and Economic Value of Long-Acting Preexposure Prophylaxis for South African Women at High-Risk for HIV Infection.* J Infect Dis, 2016. **213**(10): p. 1523-31.

5. Pretorius, C., et al., *Evaluating the cost-effectiveness of pre-exposure prophylaxis (PrEP) and its impact on HIV-1 transmission in South Africa.* PLoS One, 2010. **5**(11): p. e13646.

6. Smith, J.A., et al., *Maximising HIV prevention by balancing the opportunities of today with the promises of tomorrow: a modelling study.* Lancet HIV, 2016. **3**(7): p. e289-96.

7. Hallett, T.B., et al., *Optimal uses of antiretrovirals for prevention in HIV-1 serodiscordant heterosexual couples in South Africa: a modelling study.* PLoS Med, 2011. **8**(11): p. e1001123.

8. Jewell, B.L., et al., *Estimating the cost-effectiveness of pre-exposure prophylaxis to reduce HIV-1 and HSV-2 incidence in HIV-serodiscordant couples in South Africa.* PLoS One, 2015. **10**(1): p. e0115511.

9. Cremin, I., et al., *The new role of antiretrovirals in combination HIV prevention: a mathematical modelling analysis.* AIDS, 2013. **27**(3): p. 447-58.

10. Long, E.F. and R.R. Stavert, *Portfolios of biomedical HIV interventions in South Africa: a cost-effectiveness analysis.* J Gen Intern Med, 2013. **28**(10): p. 1294-301.

11. Chiu, C., et al., *Designing an optimal HIV programme for South Africa: Does the optimal package change when diminishing returns are considered?* BMC Public Health, 2017. **17**(1): p. 143.

12. Alsallaq, R.A., et al., *The potential impact and cost of focusing HIV prevention on young women and men: A modeling analysis in western Kenya.* PLoS One, 2017. **12**(4): p. e0175447.

13. Cremin, I. and T.B. Hallett, *Estimating the range of potential epidemiological impact of pre-exposure prophylaxis: run-away success or run-away failure?* AIDS, 2015. **29**(6): p. 733-8.

14. Anderson, S.J., et al., *Maximising the effect of combination HIV prevention through prioritisation of the people and places in greatest need: a modelling study.* Lancet, 2014. **384**(9939): p. 249-56.

15. Cremin, I., et al., *PrEP for key populations in combination HIV prevention in Nairobi: a mathematical modelling study.* Lancet HIV, 2017. **4**(5): p. e214-e222.

16. Nichols, B.E., et al., *Cost-effectiveness of pre-exposure prophylaxis (PrEP) in preventing HIV-1 infections in rural Zambia: a modeling study.* PLoS One, 2013. **8**(3): p. e59549.

17. Nichols, B.E., et al., *Cost-effectiveness of PrEP in HIV/AIDS control in Zambia: a stochastic league approach.* J Acquir Immune Defic Syndr, 2014. **66**(2): p. 221-8.

18. Cremin, I., et al., *Seasonal PrEP for partners of migrant miners in southern Mozambique: a highly focused PrEP intervention.* J Int AIDS Soc, 2015. **18**(4 Suppl 3): p. 19946.

19. Mitchell, K.M., et al., *Modelling the impact and cost-effectiveness of combination prevention amongst HIV serodiscordant couples in Nigeria.* AIDS, 2015. **29**(15): p. 2035-44.

20. Ying, R., et al., *Cost-effectiveness of pre-exposure prophylaxis targeted to high-risk serodiscordant couples as a bridge to sustained ART use in Kampala, Uganda.* J Int AIDS Soc, 2015. **18**(4 Suppl 3): p. 20013.

21. Stover, J., et al., *How can we get close to zero? The potential contribution of biomedical prevention and the investment framework towards an effective response to HIV.* PLoS One, 2014. **9**(11): p. e111956.

22. McGillen, J.B., S.J. Anderson, and T.B. Hallett, *PrEP as a feature in the optimal landscape of combination HIV prevention in sub-Saharan Africa.* J Int AIDS Soc, 2016. **19**(7(Suppl 6)): p. 21104.

23. McGillen, J.B., et al., *Optimum resource allocation to reduce HIV incidence across sub-Saharan Africa: a mathematical modelling study.* Lancet HIV, 2016. **3**(9): p. e441-e448.

24. Verguet, S., M. Stalcup, and J.A. Walsh, *Where to deploy pre-exposure prophylaxis (PrEP) in sub-Saharan Africa?* Sex Transm Infect, 2013. **89**(8): p. 628-34.

25. Abbas, U.L., R.M. Anderson, and J.W. Mellors, *Potential impact of antiretroviral chemoprophylaxis on HIV-1 transmission in resource-limited settings.* PLoS One, 2007. **2**(9): p. e875.

26. Price, J.T., et al., *Cost-Effectiveness of Pre-exposure HIV Prophylaxis During Pregnancy and Breastfeeding in Sub-Saharan Africa.* J Acquir Immune Defic Syndr, 2016. **72 Suppl 2**: p. S145-53.
